# Prevalence of neck pain in subjects with metabolic syndrome - a cross-sectional population-based study

**DOI:** 10.1186/1471-2474-11-171

**Published:** 2010-07-30

**Authors:** Pekka Mäntyselkä, Hannu Kautiainen, Mauno Vanhala

**Affiliations:** 1 School of Medicine, Primary Health Care, University of Eastern Finland, Kuopio, Finland and Unit of Primary Health Care, Kuopio University Hospital, Kuopio, Finland; 2 ORTON Foundation, Helsinki, Finland; 3 School of Medicine, Primary Health Care, University of Eastern Finland, Kuopio, Finland and Unit of Family Practice, Central Hospital of Middle Finland, Jyväskylä, Finland

## Abstract

**Background:**

Metabolic syndrome (MetS) is increasingly common. Obesity has been suggested to associate with neck pain but prevalence of neck pain in subjects with MetS has not been studied. Aim of this study was to analyse the association between MetS and neck pain.

**Methods:**

The study population consisted of 1294 middle-aged subjects in Pieksämäki, Finland. A total of 399 males and 500 females participated (69%). The mean age of both males and females was 46 years. Clinical and biochemical measurements were taken. The participants filled out a standard questionnaire. Psychological distress was assessed with the 12-item General Health Questionnaire (GHQ-12). Neck pain was defined as neck pain perceived daily. MetS was defined using National Cholesterol Education Program (NCEP) criteria. Statistical comparisons between the groups were performed using a bootstrap-type t-test or Chi-Square test. Risk ratios of having neck pain were calculated using generalised linear models with age, smoking, alcohol use, exercise and GHQ-12 score as covariates.

**Results:**

The prevalence of MetS was 33% in males and 29% in females. Neck pain was present in 11% (N = 42) of males and 19% (N = 93) of females (P < 0.001). The prevalence of neck pain was 7.9% (95% CI, 4.9% to 12%) among male subjects without MetS and 16% (95% CI, 10% to 23%) among those with MetS. The respective proportions among females were 16% (95% CI, 12% to 20%) and 25% (95% CI, 18% to 33%). The multivariate analysis showed an increased risk of neck pain in males with MetS (RR 2.1, 95% CI, 1.2 to 3.7, P = 0.010) and in females with MetS (RR 1.5, 95% CI, 1.0 to 2.1, P = 0.040).

**Conclusions:**

MetS was associated with neck pain. This association was stronger in males, but the prevalence of neck pain was higher in females. Prospective studies should explore the potential causal association between neck pain and MetS and the potential common background factors of neck pain and MetS.

## Background

Metabolic syndrome (MetS) has become increasingly common worldwide [1]. Metabolic syndrome (MetS) is a cluster of risk factors defined by high fasting glucose and triglycerides, low HDL cholesterol, high blood pressure, and abdominal obesity that increases the risk for cardiovascular diseases, type 2 diabetes mellitus, and all-cause mortality [2-4]. The prevalence of MetS in the US population is approximately 35% [5]. In Eastern Finland the corresponding prevalence has been found to be 37% [6]. Neck pain is also a common symptom among the middle-aged population. In a large Finnish population-based study, 24% of men and 37% of women aged at least 30 years had suffered from neck pain during the preceding month [7].

There are few studies in which the prevalence of pain has been assessed in subjects with MetS. In one study females with chronic pain from fibromyalgia were at an increased risk of MetS [8]. Another study found that subjects with metabolic syndrome were more likely to have problems with pain symptoms [9]. It has been suggested that stress is related to both MetS and neck pain [10,11]. Low physical activity has been found to be associated with MetS [12] and musculoskeletal pain [13]. Some studies have found an association between obesity and neck pain [14,15]. Because visceral obesity is one of the main features of MetS, it could be proposed that MetS is also related to neck pain. It has been speculated that both MetS and persistent chronic pain syndromes are related to hypothalamus-pituitary-adrenal stress axis dysfunction [16,17]. Therefore, it could be expected that the prevalence of neck pain is elevated in subjects with MetS. Thus, if there were common features in the background of these disorders, we hypothesized that neck pain is more prevalent among subjects with MetS than among those without MetS. In this study we aimed to analyse the prevalence of neck pain in subjects with MetS.

## Methods

The original study population consisted of middle-aged, Finnish subjects (N = 1294) born in 1942, 1947, 1952, 1957 and 1962 (the entire age groups, age range at the baseline being 35-56 years) in Pieksämäki, a town in eastern Finland. Altogether 923 of 1294 subjects (71.3%) participated in the initial examination in 1997-98. The mean age of the study population was 46 years. Body mass index was 26.7 kg/m^2 ^in men and 26.3 kg/m^2 ^in women (P = 0.15) [18]. For the present study, complete data were available for 399 males and 500 females who participated. The mean age of both males and females was 46 years. The prevalence of neck pain was analysed stratified by sex because prevalence of MetS in Finland is more prevalent among males than females [5,18]. Further, neck pain is more common among females than among males [7] and the factors associating with neck pain may be different in females [19].

Metabolic syndrome was defined based on the criteria proposed by the NCEP [20]. In the evaluation of MetS, we used the modified National Cholesterol Education Program - Adult Treatment Panel III criteria (NCEP-ATP III), with the 5.6 mmol/l blood glucose cut-off point [21]. NCEP defines MetS as having three or more of the following criteria: 1) fasting serum glucose of 5.6 mmol/l or higher, 2) serum triglycerides of 1.7 mmol/l or higher, 3) serum high-density lipoprotein (HDL) cholesterol < 1.03 mmol/l in men or < 1.29 mmol/l in women, 4) blood pressure of 130/85 mmHg or higher and 5) waist circumference > 102 cm in men or > 88 cm in women. Serum triglycerides, cholesterol, high-density lipoprotein (HDL) cholesterol, glucose and high sensitivity C-reactive protein (hs-CRP) were measured [18]. Blood pressure was measured twice at 5 min intervals; the readings from the second measurement were used in the analyses. Body mass index (BMI) was calculated as kg/m^2^. Persons who smoked at least once a week were labelled as current smokers. Current use of alcohol was defined as alcohol consumption at least once a year. Leisure time physical activity was defined as physical exercise that lasted at least 30 minutes per session and caused sweating. There were three categories of physical exercise: low activity (1-2 times a month or less), moderate activity (1-3 times a week) and high activity (more than 3 times a week).

Psychological distress was assessed with the 12-item General Health Questionnaire (GHQ-12) [22]. The four-point response scale was rated as follows: presence of symptom: not at all = 0, same as usual = 0, more than usual = 1, much more than usual = 1. The study subjects were allocated into three groups according to their scores (0 points, 1-2 points and 3-12 points).

Neck pain was assessed by asking about neck pain during the preceding month. The presence of neck pain was dichotomized: (0) no neck pain or neck pain only occasionally and (1) daily or almost daily neck pain. Hence, in this study we regarded neck pain as daily or almost daily occurring neck pain.

Statistical comparisons between the groups were performed using a bootstrap-type t-test or Chi-Square test. Risk ratios (RR) of having neck pain were estimated by a generalised linear model with the binomial family, log link, and robust standard error. In these analyses age, smoking, alcohol use, exercise and GHQ-12 score were used as covariables in order to control for factors that potentially influence the relationship between neck pain and MetS. It has been found that smoking can affect the impact of neck pain, but moderate alcohol use may have a protective effect [23]. Low physical activity has been found to be associated with MetS and musculoskeletal pain [12,13]. Psychological distress has been suggested to be related to both MetS [24] and neck pain [19]. Confidence intervals (CI) for the percentages were obtained by exact (Clopper-Pearson) methods. Stata statistical software, release 10 (StataCorp, College Station, Texas) was used for the analyses.

This study was approved by the Ethics Committee of the Kuopio University Hospital. All the participants gave an informed consent.

## Results

The mean age of the male study subjects was 46.1 years (SD 6.3 years) and that of the females was 45.9 years (SD 6.2 years) (P = 0.69). MetS was present in 132 men (33%) and 146 women (29%). Neck pain was present in 18.6% (N = 93) of the females and 10.5% (N = 42) of the males (P < 0.001). The subjects with neck pain were slightly but not significantly older than those who had no neck pain. The mean age of the male subjects without neck pain was 46.0 (SD 6.3) years and that of the subjects with neck pain was 47.0 years (SD 6.3) (P = 0.30). The corresponding mean ages of the females were 45.6 years (SD 6.2) and 47.0 years (SD 6.2) (P = 0.06).

The clinical data are presented in Table 1. Compared with the subjects who had no neck pain, MetS was more prevalent among males and females who suffered from neck pain. The subjects with neck pain more often had antihypertensive medication than did those without neck pain. Alcohol use was inversely related to neck pain in females but not in males. BMI and waist circumference were associated with neck pain in males but not in females. The GHQ-12 score was significantly higher for males and females with neck pain than for those who had no neck pain. There were no significant differences in smoking, physical activity, blood pressure, lipids, glucose and CRP levels between the subjects with neck pain and without neck pain.

**Table 1:** Clinical data in males and females according to neck pain status.

	MALES (N = 399)			FEMALES (N = 500)		
	No Neck Pain (N = 357)	Neck Pain (N = 42)	P-value	No Neck Pain (N = 407)	Neck Pain (N = 93)	P-value
Metabolic syndrome, N (%)	111 (31)	21 (50)	0.014	110 (27)	36 (39)	0.025
Diabetes, N (%)	30 (8.4)	2 (4.8)	0.41	23 (5.7)	3 (3.2)	0.34
Antihypertensive medication, N (%)	33 (9)	8 (19)	0.048	29 (7)	14 (15)	0.014
Lipid-lowering medication, N (%)	11 (3.1)	1 (2.4)	0.80	5 (1.2)	1 (1.1)	0.90
Smoking, N (%)	121 (34)	15 (36)	0.81	87 (21)	25 (27)	0.25
Alcohol use, N (%)	312 (87)	35 (83)	0.46	332 (82)	67 (72)	0.039
Physical activity* high, N (%)	49 (14)	5 (12)	0.94	59 (14)	11 (12)	0.85
Diastolic blood pressure, mmHg, mean (SD)	83.5 (9.9)	85.3 (11)	0.30	79.7 (9.3)	79.5 (10)	0.85
Systolic blood pressure, mean, mmHg (SD)	136.1 (17)	141.0 (19)	0.12	131.2 (17)	131.3 (19)	0.94
Body Mass Index, kg/m^2^, mean (SD),	26.5 (3.4)	28.0 (5.5)	0.012	26.4 (5.4)	26.6 (4.6)	0.74
Waist circumference, cm, mean (SD)	93 (10)	98 (14)	0.0081	83 (12)	85 (13)	0.19
S-cholesterol^† ^, mmol/l, mean (SD)	5.8 (1.0)	6.1 (1.3)	0.20	5.6 (0.9)	5.8 (1.2)	0.26
S-HDL-cholesterol^#^, mmol/l, mean (SD)	1.3 (0.3)	1.3 (0.3)	0.86	1.5 (0.3)	1.5 (0.3)	0.57
S-triglyserides, mmol/l mean (SD)	1.6 (1.0)	2.2 (2.5)	0.16	1.2 (0.6)	1.3 (0.7)	0.11
P-glucose^‡^, mmol/, mean (SD)	6.0 (0.9)	5.9 (0.6)	0.54	5.6 (0.6)	5.7 (0.5)	0.75
hs-CRP^¶^, mg/l, mean (SD)	1.5 (2.8)	1.8 (1.8)	0.41	1.7 (2.3)	1.8 (1.7)	0.65
GHQ-12^§ ^sum, mean (SD)	1.3 (2.4)	2.5 (3.1)	0.002	1.5 (2.7)	3.6 (3.9)	< 0.001

* Leisure time physical activity was defined as physical exercise that lasted at least 30 minutes per session and caused sweating. There were three categories: low activity (1-2 times a month or less), moderate activity (1-3 times a week) and high activity (more than 3 times a week).

†S-cholestero l = Serum cholesterol

# S-HDL-cholesterol = Serum high density lipoprotein cholesterol

‡ P-glucose = Plasma glucose

¶ hs-CRP = High sensitivity C-reactive protein

§ GHQ-12 = 12-item General Health Questionnaire

**Figure 1:**
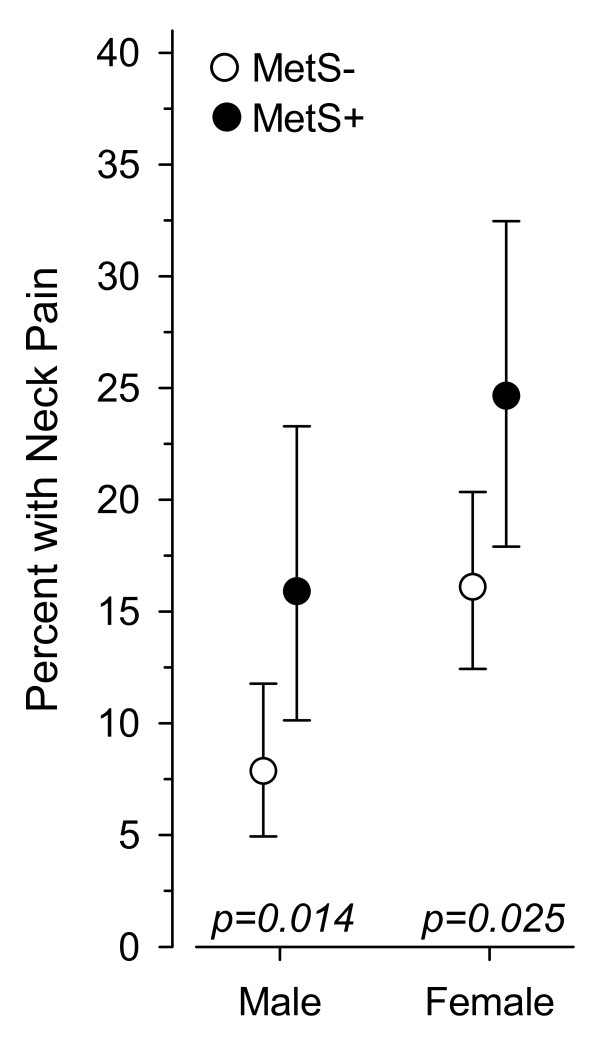
**Prevalence of neck pain in females and males with and without metabolic syndrome (MetS)**.

Compared with the subjects without metabolic syndrome, both male and female subjects with metabolic syndrome more often had neck pain (Figure 1 and Table 1). The prevalence of neck pain was 7.9% (95% CI, 4.9% to 12%) among male subjects without MetS and 16% (95% CI, 10% to 23%, P = 0.014) among those with MetS. The respective proportions were 16% (95% CI, 12% to 20%) and 25% (95% CI, 18% to 33%, P = 0.025) among females.

In the univariate analysis MetS was associated with neck pain (a crude risk for males and females, RR = 1.6 (95% CI, 1.2 to 2.2)). The risk ratio was 2.0 (95% CI, 1.1 to 3.6, P = 0.015) in males and 1.5 (95% CI, 1.1 to 2.2, P = 0.024) in females. The multivariate analysis (Table 2) showed an increased risk of neck pain for male subjects with MetS (RR 2.1, 95% CI, 1.2 to 3.7, P = 0.010). Also the GHQ score indicated an increased risk of neck pain in males. The RR of a GHQ score of 3 or more was 3.2 (95% CI, 1.7 to 6.1). Female subjects with MetS had a risk of neck pain (RR 1.5, 95%, 1.0 to 2.1, P = 0.040). In females a GHQ score was associated with neck pain. The risk ratio of a GHQ score of 1 to 2 was 2.7 (95% CI, 1.7 to 4.4, P < 0.001), and for a GHQ score of 3 or more the RR was 3.0 (95% CI, 1.9 to 4.6, P < 0.001). Age, smoking, alcohol use and physical activity were associated with neck pain in neither males nor females.

**Table 2:** Risk ratios of neck pain in males and females.

	MALES		FEMALES	
	Risk Ratio (95% CI)	P-Value	Risk Ratio (95% CI)	P-Value
Metabolic syndrome	2.09 (1.19 - 3.67)	0.01	1.46 (1.02 - 2.10)	0.04
Age	1.00 (0.96 - 1.05)	0.89	1.02 (0.99 - 1.05)	0.25
Physical Activity*				
Moderate	0.89 (0.47 - 1.66)	0.70	0.97 (0.66 - 1.44)	0.89
High	0.73 (0.29 - 1.87)	0.53	0.70 (0.38 - 1.29)	0.25
Psychologic distress				
Moderate (GHQ-12^§^; 1-2)	1.64 (0.77 - 3.49)	0.20	2.74 (1.69 - 4.45)	< 0.001
High (GHQ-12; 3-12)	3.20 1.67 - 6.13)	< 0.001	2.98 (1.91 - 4.64)	< 0.001
Smoking	1.02 (0.57 - 1.83)	0.94	1.32 (0.90 - 1.93)	0.16
Alcohol use	0.75 (0.36 - 1.56)	0.44	0.73 (0.50 - 1.07)	0.11

Results of multivariate analysis.

* Leisure time physical activity was defined as physical exercise that lasted at least 30 minutes per session and caused sweating. There were three categories: low activity (1-2 times a month or less), moderate activity (1-3 times a week) and high activity (more than 3 times a week).

§ GHQ-12 = 12-item General Health Questionnaire

## Discussion

This study showed that males and females with metabolic syndrome have an increased prevalence of neck pain. This association was stronger in males, but the prevalence of neck pain was higher in females. In accordance with previous studies, psychological distress was associated with neck pain especially in females. Although psychological distress was taken into account, MetS was statistically associated with neck pain.

BMI was higher and waist circumference larger in males with neck pain. Previous studies have suggested that obesity is a risk factor of neck pain [14,15]. Subjects with MetS are often obese and waist size is among the criteria of MetS. However, BMI and waist size were similar in females regardless of neck pain. Therefore, it is not plausible that the association between MetS and neck pain is related solely to obesity. Compared with females, males with neck pain had higher cholesterol and triglyceride levels and a higher BMI. Psychological distress was associated with neck pain in both genders. However, a lower lever of distress was associated with neck pain in females, but in males only severe distress had that association. In general, the level of psychological distress was higher among females than among males. According to a large population-based study, concurrent psychological distress is more prevalent among females [19].

The population of the present study was from a limited area in Finland, and the number of subjects with neck pain was not very large. Therefore, our results should be regarded as preliminary and they should be generalized cautiously to other populations. The assessment of neck pain was based on self-reports and we did not more deeply assess the diagnostic features of these symptoms, which also is a potential limitation of our study.

One background hypothesis for the connection between neck pain and MetS found in this study is that there is a common factor resulting in the development of both neck pain and MetS. Two such factors could be stress and physical inactivity. Stress has been suggested to be a risk factor of MetS [10]. A recent study has suggested that workers with neck, shoulder, or back pain have elevated levels of stress-related biomarkers [11]. Further, it can be speculated that neck pain is an indicator of stress. A recent study has shown that in a specified population, physical inactivity is a risk of MetS, whereas perceived stress was not associated with MetS [25]. The association between development of MetS and low physical inactivity has also been shown in a previous study [12]. A large epidemiological follow-up study indicated that physical inactivity is related to chronic musculoskeletal complaints [13]. It has been suggested that chronic musculoskeletal pain is associated with cardiovascular-related mortality [26]. Hence, physical inactivity may be an intervening factor between MetS and neck pain. Further studies with a longitudinal setting could explore the potential causal association between neck pain and MetS as well as the potential common background factors of neck pain and MetS.

## Conclusions

MetS was associated with neck pain. This association was stronger in males but the prevalence of neck pain was higher in females. Prospective studies focusing on the causal relationship between neck pain and metabolic syndrome are needed.

## Competing interests

The authors declare that they have no competing interests.

## Authors' contributions

PM participated in the analysis of data and in design of the manuscript and drafted the manuscript. HK analysed the data and participated in design of the manuscript. MV conceived of the study and participated in its design, analysis and coordination and helped to draft the manuscript. All authors read and approved the final manuscript.

## Pre-publication history

The pre-publication history for this paper can be accessed here:

http://www.biomedcentral.com/1471-2474/11/171/prepub
